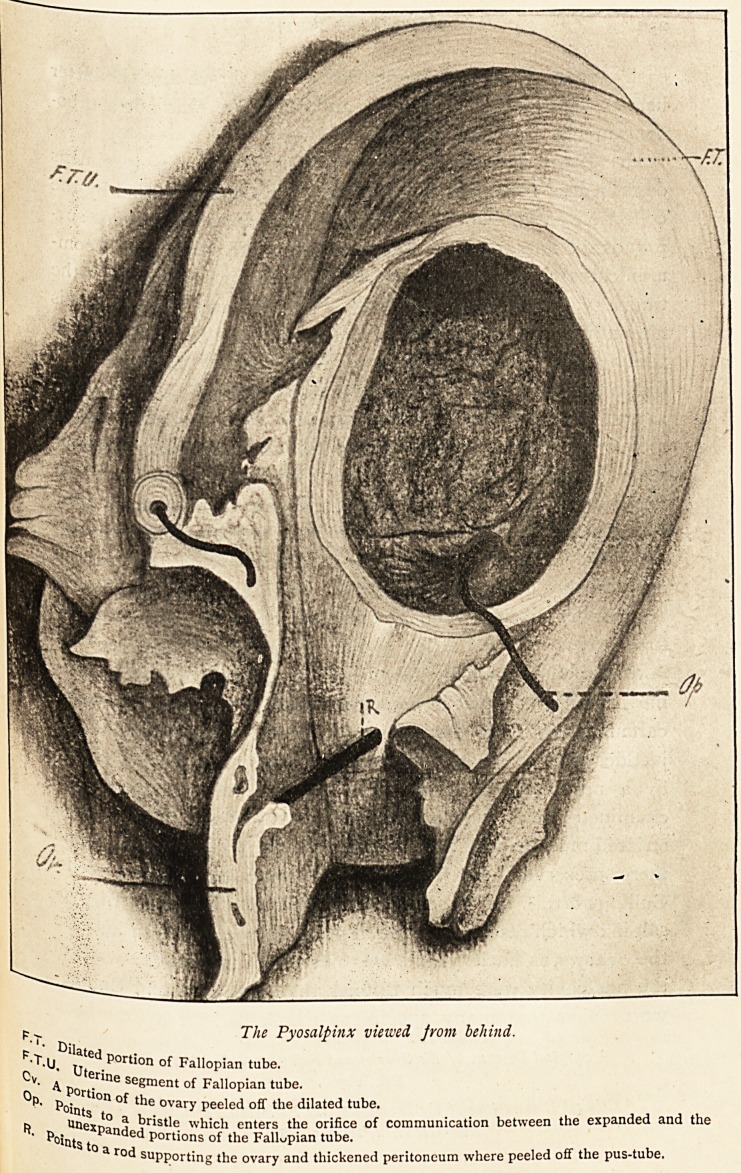# A Case of Laparotomy in Which a Large Pyosalpinx Simulating a Suppurating Tubo-Ovarian Cyst Was Removed

**Published:** 1898-09

**Authors:** J. Lacy Firth

**Affiliations:** Assistant-Surgeon to the Bristol General Hospital


### A CASE OF LAPAROTOMY IN WHICH A LARGE PYOSALPINX SIMULATING A SUPPURATING TUBO-OVARIAN CYST WAS REMOVED.

BY

J. Lacy Firth, M.S. Lond., F.R.C.S. Eng.,

*Assistant-Surgeon to the Bristol General Hospital.*

---

The specimen of pyosalpinx of which a drawing is here reproduced, and which was removed by laparotomy from a patient in the Bristol General Hospital, presents some features of special interest. It includes practically the whole of the right broad ligament containing a large cyst, and the Fallopian tube and ovary. The cyst is of about the size and shape of a cocoa-nut. It has thick walls and rather resembles a hypertrophied urinary bladder in appearance. The Fallopian tube is much thickened and elongated, but not tortuous, and lies closely applied to the outer surface of the cyst. From the uterus the tube takes an inverted U-shaped course, with the cyst lodged in its concavity. One limb of the U is formed by the uterine half of the tube, which lies upon the upper and anterior part of the cyst; the other limb, by the distal half attached to the lower and posterior part of the cyst. About one-fourth of the circumference of the cyst is not embraced by the tube, and that is the portion which originally lay adjacent to the uterus. Distally the tube appears to end on the cyst-wall in the middle of its lower surface, and in what appears to be the last inch it is widened out and flattened; but neither at this nor at any other place can fimbriæ be detected.

The cyst had a polished and dark-red external surface and was free from adhesions. Its internal surface was, and still is, smooth also, though slightly uneven, the appearance suggesting a lining of mucous membrane. An oval aperture, measuring about a third of an inch in its long axis, leads from the cavity of the cyst into the lumen of the Fallopian tube, and is situated at the place where the flattened portion of the tube appears to end on the external surface. In the fresh state of the specimen the opening would have been large enough to admit the end of an ordinary cedar pencil. From this aperture to its uterine end the tube is patent, admitting in the latter situation a fine bristle. The cyst contained about a pint of thick pus.

When the tumour was examined at the conclusion of the operation, there was found on its external surface a small cyst about the size of a split pea and only very slightly raised above the general level. This was low down posteriorly, near where the uterus had been. This small cyst was the only structure suggesting the presence of ovarian tissue which could be seen without dissecting the specimen. At a later date more ovarian tissue was found in the same region by making incisions into the main cyst-wall, and it was further found that this tissue could be easily peeled off the deeper layers of the wall, leaving on the latter a smooth surface. Not only could the flattened remnants of the ovary be peeled off in this way, but also a layer of tissue continuous with them on all sides. This separable layer, which is represented in the drawing as it appeared when partially turned to the right and left after making an incision through it and through the ovary, is obviously the thickened peritoneum and cellular tissue of the broad ligament, and no doubt it could be peeled off the whole of the cyst. At the time the separation was made the specimen had been hardened for three weeks in spirit, which I believe made the separation easier.

Until the separability of the ovary from the deeper layers of the cyst-wall had been discovered, the specimen had been regarded as one probably of suppurating tubo-ovarian cyst or ovarian hydrocele, and I think everyone will admit that the resemblance it bore to specimens of the class mentioned was very great.

A tubo-ovarian cyst is usually understood to be an ovarian cyst, generally unilocular, which communicates by a considerable aperture with the adherent fimbriated extremity of a dilated Fallopian tube, a condition first carefully described by Richard in 1853.[1] The fact that in my specimen the ovary was fairly

[1] *Mém. Soc. de Chir. de Par.*, 1851–53, iii. 121; quoted by Griffith, *Tr. Obst. Soc. Lond.*, 1888, xxix. 274.

*The Pyosalpinx viewed from behind.*

F.T. Dilated portion of Fallopian tube.

F.T.U. Uterine segment of Fallopian tube.

Cv. A portion of the ovary peeled off the dilated tube.

Op. Points to a bristle which enters the orifice of communication between the expanded and the unexpanded portions of the Fallopian tube.

R. Points to a rod supporting the ovary and thickened peritoneum where peeled off the pus-tube.

easily separable from the suppurating cyst, shows that the latter had an independent formation, and, therefore, was not a tubo-ovarian cyst as defined above.

Mr. Bland Sutton [1] believes that many of the specimens described as tubo-ovarian cysts are really cysts formed by the collection of fluid in a peritoneal tunic which sometimes surrounds the human ovary, forming a complete ovisac which communicates with the Fallopian tube. The tunic is analagous to the tunica vaginalis of the testicle. In rats and mice there is normally a complete ovisac of this kind, and specimens exist in which hydroceles and abscesses have been formed by their distension with fluid. In the hyena and tigers there are ovisacs almost complete, communicating with the peritoneal cavity merely by a small fringed orifice, which might easily become sealed up by inflammation. But in ovarian hydrocele the ovary forms an integral part of the cyst-wall, and is inseparable from it. My specimen is not one of suppurating ovarian hydrocele therefore. Nor is it an example of a tubo-ovarian abscess, for again in that case the ovary would be inseparable, the abscess being in its substance though communicating with the Fallopian tube. It is in fact one of pyosalpinx in which the distended portion of the tube is acutely flexed upon the other part, and the most interesting point in connection with it is that it required a careful dissection to distinguish it from the diseased conditions just described. That it is one of pyosalpinx is further shown by a microscopical examination of its wall. A section so examined shows the presence of a well-defined layer of non-striated muscle in it. The lining epithelium was only here and there preserved. It was a single layer of flattened cells. Cullingworth [2] has described and figured a specimen of pyosalpinx which resembles mine in many respects; but in his case the ovary was healthy and was not removed, and the walls of the pus-cavity were much thinner than in my case. Cullingworth says that many specimens which had for a time been

[1] *Surgical Diseases of the Ovaries and Fallopian Tubes*, New [2nd] Ed., 1896.

[2] *Clinical Illustrations of the Diseases of the Fallopian Tubes and of Tubal Gestation*, 1895, p. 28.

thought to be tubo-ovarian cysts, have on further examination been found to be dilated tubes. I have shown that such was the case with this specimen, and so incidentally illustrated the adage that, "history repeats itself."

My specimen was obtained from a woman, aged 39, an inmate of the Bristol General Hospital. She had been married seventeen years, but had had no children and no miscarriages. Menstruation was regular, but always associated with pain the day before and the first day of the period. This pain she had always had since menstruation was established. Her illness began with sudden abdominal pain in the hypogastric and right iliac regions. The pain seized her one Sunday morning when sitting quietly by the fire drinking tea. Menstruation had ceased the day before. This was five weeks before her admission to the Hospital and her operation. The pain never left her from its first onset, and she had kept her bed. The abdomen had gradually become distended, especially in the lower part. She had had vomiting almost daily, and had been feverish. Micturition had been painful and diarrhœa frequent. Her state on April 11th, the date of the operation, was not promising. The pulse was 120 and very weak, the temperature 101.5° F. She had a good deal of bronchitis, and great abdominal pain and tenderness. She was a stout person, but anæmic. The abdomen was tense, especially in the right iliac and hypogastric regions, where a very tender and tense swelling was palpable, dull on percussion and extending upwards nearly to the umbilical level. The borders were ill-defined. The lower part of this swelling could be reached from the right lateral fornix on bimanual vaginal examination. I opened the abdomen in the median line, and immediately came upon the cystic swelling with the thickened tube passing over it. I thought it must be a suppurating broad ligament cyst in the first instance, and made an attempt to shell it out after tapping. Finding that impossible, I removed the mass in its entirety by ligaturing the broad ligament in succesive segments at the uterine and pelvic ends. The patient's convalescence was slow. The bronchitis was very troublesome for the first week. On the third day I removed a Keith's drainage tube which I had used. Cystitis developed later. Six weeks after the operation she was, however, quite well and active.

I am much indebted to Mr. E. V. Foss, a medical student of the Hospital, for the excellent drawing of the specimen here reproduced.

### Progress of the Medical Sciences.

## Figures and Tables

**Figure f1:**